# Effectiveness of Home-Based Pain-Free Exercise versus Walking Advice in Patients with Peripheral Artery Disease: A Randomized Controlled Trial

**DOI:** 10.3390/mps4020029

**Published:** 2021-05-10

**Authors:** Fabio Manfredini, Nicola Lamberti, Luca Traina, Gladiol Zenunaj, Chiara Medini, Giovanni Piva, Sofia Straudi, Roberto Manfredini, Vincenzo Gasbarro

**Affiliations:** 1 Department of Neuroscience and Rehabilitation, University of Ferrara, 44121 Ferrara, Italy; fabio.manfredini@unife.it (F.M.); nicola.lamberti@unife.it (N.L.); giovanni.piva@unife.it (G.P.); 2 Unit of Rehabilitation Medicine, University Hospital of Ferrara, 44124 Ferrara, Italy; sofia.straudi@unife.it; 3 Unit of Vascular and Endovascular Surgery, University Hospital of Ferrara, 44124 Ferrara, Italy; l.traina@ospfe.it (L.T.); g.zenunaj@ospfe.it (G.Z.); c.medini@ospfe.it (C.M.); vincenzo.gasbarro@unife.it (V.G.); 4 Department of Medical Sciences, University of Ferrara, via Fossato di Mortara 46, 44121 Ferrara, Italy

**Keywords:** peripheral artery disease, exercise therapy, rehabilitation, mobility, randomized-controlled trial

## Abstract

Exercise therapy in the intermediate stages of peripheral artery disease (PAD) represents an effective solution to improve mobility and quality of life (QoL). Home-based programs, although less effective than supervised programs, have been found to be successful when conducted at high intensity by walking near maximal pain. In this randomized trial, we aim to compare a low-intensity, pain-free structured home-based exercise (SHB) program to an active control group that will be advised to walk according to guidelines. Sixty PAD patients aged > 60 years with claudication will be randomized with a 1:1 ratio to SHB or Control. Patients in the training group will be prescribed an interval walking program at controlled speed to be performed at home; the speed will be increased weekly. At baseline and after 6 months, the following outcomes will be collected: pain-free walking distance and 6-min walking distance (primary outcome), ankle-brachial index, QoL by the VascuQoL-6 questionnaire, foot temperature by thermal camera, 5-time sit-to-stand test, and long-term clinical outcomes including revascularization rate and mortality. The home-based pain-free exercise program may represent a sustainable and cost effective option for patients and health services. The trial has been approved by the CE-AVEC Ethics Committee (898/20). Registration details: Clinicaltrials.gov NCT04751890 [Registered: 12 February 2021].

## 1. Introduction

Peripheral artery disease (PAD) of the lower limbs is an underestimated pathology that is highly prevalent in elderly individuals [1]. At an intermediate stage, the symptom of intermittent claudication, defined as cramping pain caused by insufficient oxygen delivery to the working muscles, reduces walking ability. PAD, which, in a percentage of cases, may evolve to critical limb ischemia requiring revascularization and amputation, is primarily associated with a high risk of cardiovascular events [1]. Early management of PAD may reduce patients’ disability and functional decline as well amputations and cardiovascular events. Exercise therapy is recommended as the initial treatment for its effectiveness on the walking disability, with lower morbidity, mortality and healthcare costs compared to endovascular or surgical revascularization [1]. In particular, supervised exercise therapy (SET) programs performed at hospitals are recommended [1,2], as they can significantly improve exercise capacity in patients both with and without claudication and are more effective than unsupervised programs [1,3,4,5,6]. SET, carried out in a hospital under the supervision of qualified healthcare providers, is based on intermittent bouts of treadmill walking (30-45 min per session) until moderate to maximum claudication, alternating with periods of rest, performed at least three times/week for 12–24 weeks [1,5,6]. SET programs have shown superior effectiveness compared to endovascular revascularization [1,7] and have the advantages of persistent benefits [1,8], a favorable risk/benefit ratio, safety and few absolute contraindications [1,2,6,8,9,10]. Unfortunately, SET programs are underutilized [1], not widely available, differently implemented [3] and not attractive to patients due to issues of reimbursement, transportation, awareness, motivation and eligibility [2,10]. SET also has also shown a poor impact on patients’ lifestyles in terms of low long-term adherence rates and high variability in rehabilitative outcomes [5,6,9,11] with a high nonresponse rate (37.5%) [12] and lower benefits paradoxically observed in patients with more severe disability [5].

The recently proposed structured home-based (SHB) exercise programs, carried out in the personal setting of the patient [1,6,13], represent an alternative option [14,15], reducing the barriers to exercise, such as the availability of facilities and funding for SET programs [14,15], transportation time and costs for patients and families [4]. These programs make use of behavioral change techniques, health coaching, and tools for self-monitoring of home exercise sessions [9,16,17]. Appointed healthcare providers prescribe structured programs that are generally based on walking sessions progressively increasing from 10 to 50 min at a self-selected pace or to maximal ischemic leg pain, 3-5 times per week [9,16,17]. In some research, SHB exercise showed significant benefits [5]; in other studies, it improved overground walking only when carried out at high intensity [18,19]; but a trial based on coaching, telephone calls and use of wearable activity monitors showed no improvement [20]. SHB is even less effective than SET but is still recommended with a Class IIa Level of Evidence in the clinical practice guidelines for PAD [1,5]. A patient-centered SHB model, the “test in-train out” program (TiTo), was proposed by Manfredini et al. [21,22,23,24,25] when SHB programs were not yet available. This model contemplates the execution of pain-free exercise completely at home, along with check-ups at the hospital approximately once a month for therapy and adherence evaluation, functional assessment and personalized prescription of exercise.

This patient-oriented program, which is associated with significant improvements in performance and quality of life at discharge, low costs and favorable long-term clinical outcomes [21,22,23,24,25,26,27,28,29], was successfully adapted to dialysis patients [30,31] and stroke survivors [32]. Despite its effectiveness in previous studies, the TiTo program was never compared in a randomized controlled trial to the “usual-care” treatment of PAD by advising a walking regimen as suggested by the guidelines. We hypothesize that the TiTo-SHB exercise program may be superior to the usual care treatment in patients with claudication.

The present trial aims to verify the superiority of the TiTo-SHB model compared to walking advice on physical functioning, hemodynamics and quality of life in a cohort of claudication patients.

## 2. Methods and Analysis

### 2.1. Trial Design

This study protocol is reported according to the Standard Protocol Items: Recommendations for Interventional Trials (SPIRIT) guidelines [33]. The study is designed as a single-center parallel-group randomized controlled trial.

### 2.2. Study Setting

The study will take place, starting from 2021, at Azienda Ospedaliero-Universitaria di Ferrara at the Operative Units of Vascular and Endovascular Surgery and Rehabilitation Medicine. An indoor temperature-controlled environment at the Unit of Rehabilitation Medicine will be identified for the outcome measurements.

### 2.3. Eligibility Criteria

Inclusion criteria: Male and female aged > 60 years old; PAD at Leriche-Fontaine stage IIa or IIb, previously diagnosed by clinical and instrumental examination at the Department of Vascular Surgery according to the published standard [1]. An Echo-Color-Doppler will be performed to assess abdominal aorta and iliac arteries, common, superficial and deep femoral arteries, popliteal arteries and both tibial axes [1].

Exclusion criteria: PAD at stage I or at stage III or higher; Ankle-brachial index > 0.9 or <1.4; major cognitive disorders (identified by a Mini-Mental State Examination score < 18/30 [34]); severe uncorrected anemia (hemoglobin concentration < 10 g/dL); severe cardiorespiratory, neurological, or musculoskeletal conditions contraindicating or inhibiting exercise (e.g., major amputation, heart failure at a New York Heart Association score of III or greater); very good exercise capacity determined by a 6-min walking distance > 500 m.

### 2.4. Recruitment

During the first meeting with potential participants, a physician will explore their interest in taking part in the study. In the event of a positive response, patients will be referred for a screening visit to verify that they meet the inclusion criteria. If a patient meets all criteria, he/she will receive an information sheet on the trial as well as a consent form, and he/she will be encouraged to ask questions at will. After a minimum of three days, the patient will be contacted and asked whether he/she has decided to participate; in the event of a positive response, a meeting will be scheduled to perform the outcome measurements, and in the event of a negative response, the patient will be thanked for his/her consideration. The total numbers of screened and recruited patients will be tracked.

### 2.5. Randomization and Blinding

A team member responsible for the enrollment will generate the allocation sequence on a personal password-protected computer, with access granted only to the research coordinator and the administrator. Then, in order to prevent selection bias, patients will be randomized between the two groups through a computerized randomization stratification approach by an external administrator not involved in the trial. Participants will be stratified by their degree of PAD severity (ankle-brachial index [ABI] > or <0.5) and the presence of diabetes. The randomization scheme (1:1 ratio) will be structured in permuted blocks of 8 to ensure a similar number of participants between groups. Ultimately, each of the subjects will be assigned to receive one of two treatments: TiTo-SHB (experimental group) or walking advice as an active control (C). The participants cannot be blinded to the two interventions, as the training protocol is detailed in the information form. Once randomized, a participant will not be transferred to the other treatment group for any reason (even at the participant’s request), but the treatment may be suspended upon the patient’s request or the physician’s decision.

Researchers responsible for outcome measurement will be blinded to group allocation; unblinding will not be possible for any reason.

### 2.6. Interventions

Patients providing written informed consent will be randomized to TiTo-SHB or C.

#### 2.6.1. TiTo-SHB Group

The structured home-based TiTo walking program will be prescribed during serial visits to the hospital [21,22,23]. The program will include two 8-min sessions per day (6 days/week) of intermittent walking (1 min of exercise and 1 min of seated rest) at a prescribed speed converted into a walking cadence and followed at home using a digital metronome or a smartphone application. The walking sessions will preferably be performed overground indoors at home (e.g., in a hallway or a heated garage) to avoid the influence of weather or on a treadmill if preferred by the patient. During the trial, 3 follow-up visits (at weeks 5, 12 and 19) will be performed to evaluate patient adherence to the program and to update the exercise program, with the duration of each session remaining constant. The intensity of each walking bout will be progressively increased according to a precise scheme (Table 1). The patients will be asked to fill out a daily training record indicating completion of the exercise and any associated symptoms. Patients will be permitted to contact the rehabilitation team, composed of a physician and an exercise physiologist, by telephone throughout the study period. During the first hospital visit, a demonstrative training session will be executed with each patient.

#### 2.6.2. Control Group

Patients randomized in this group after the baseline outcome measurement session will be advised to walk as suggested by the guidelines [1,5]. In particular, a team member will recommend that patients walk for approximately 30 min at least 3 times per week; when the patient faces claudication pain, he/she will be allowed to rest and resume walking as soon as possible. A daily log will be provided to each patient to record and compile the amount of walking performed.

All patients will undergo optimization of medical therapy by the vascular surgeon. Both interventions will last 6 months.

### 2.7. Outcomes

The outcome measures will be performed at study entry prior to randomization (T0), at the end of the training program (6 months, T1) and after the 12-month follow-up (T2).

The primary outcome measure will be the 6-min walking test [35]. Patients will be asked to walk back and forth in a standard 20-m corridor with the goal of covering as much distance as possible in 6 min. Patients will be asked to report the distance at which claudication symptoms began, which will be recorded as pain-free walking distance (PFWD). When necessary, they will be allowed to stop as needed and restart whenever possible. The total distance walked, or 6-min walking distance (6MWD), will be measured. Heart rate will be continuously measured throughout the test with a heart rate monitor, and maximal oxygen consumption may be calculated according to the published formulas. The rate of perceived exertion (RPE), according to the 1-10 Borg RPE scale, will be collected at the end of the test [36]. The minimal clinically important difference for 6MWD will be set at 36 m [37].

Secondary outcomes will include the following:

The ABI will be measured according to published standards [38]. With the patients lying in a supine position, blood pressure values at the posterior tibial and dorsalis pedis arteries will be collected, along with systolic blood pressure. The ratio of the highest value measured in each leg to the pressure measured in the arm will be used to obtain the ABI values.

The Vascular Quality of Life Questionnaire-6 (VascuQoL-6) is composed of 6 questions that assess the impact of PAD on patients’ quality of life in the previous two weeks. The scores range from 6 to 24, where a higher score corresponds to a better QoL [39].

Foot temperature will be measured with an infrared thermal camera (FlirOne Pro, Flir, Milan, Italy) at three points on the dorsum of each foot: the posterior tibial artery behind the malleolus, the anterior tibial artery at the ankle, and the dorsalis pedis artery on the dorsum of the foot.

A five-time sit-to-stand test will be used to measure lower limb strength. The patient will sit with both arms folded across his/her chest, and he/she will be asked to stand and sit five times consecutively, aiming to complete them as quickly as possible. The total elapsed time will be recorded.

Long-term clinical outcomes, in terms of PAD-related revascularization, all-cause hospitalizations and survival probability, will be collected at 6, 12 and 24 months.

All outcome measurement sessions will be carried out in the same indoor temperature-controlled environment between 8.00 a.m. and 1.00 p.m.

The participant timeline of the trial is reported in Table 2.

### 2.8. Sample Size Calculation

Based on published data we observed the variations in 6MWD in TiTo-SHB program and in other trials including groups at usual care [20,23]. Considering a baseline mean 6MWD of 300 m, with 27 patients for each group, the study will have 90% power to confirm the superiority of TiTo-SHB respect to walking advice, with a superiority margin set at the minimal clinically important difference or 36 m [37]. Allowing for a possible 10% dropout rate during the trial, a sample size of 60 patients (30 per group) will be planned.

### 2.9. Data Protection and Management

A researcher blinded to the treatments will collect outcome measures and will create a password-protected electronic database containing all the data collected. Another researcher will check the completeness of the data, and he/she will randomly select some clinical records to verify that the outcomes were correctly inserted in the database.

Patients’ personal data will be treated according to the current regulations, including the 2016/679 European Union act and the D.Lgs. 30 giugno 2003, n. 196 s.m.i. Italian regulation as well as any possible adjunctive measures deemed applicable by the Italian Data Protection Authority.

### 2.10. Harms

The safe exercise procedures encompassed in this trial, along with the rigorous handling of potential harm as a local hospital policy, are expected to minimize all the potential risks. However, any potential adverse events will be recorded and treated according to the Ferrara University Hospital regulations.

### 2.11. Data Monitoring, Auditing and Interim Analyses

Owing to the single-center design of this study, a Data Monitoring Committee will not be required. Audits will be periodically scheduled with the Ferrara University Hospital clinical research office to verify the compliance of the trial with the ethical standards. The research coordinator will be in charge of the interim analysis and the final decision to stop, modify or terminate the trial.

### 2.12. Statistical Analysis

Standard analysis methods for randomized controlled trials will be employed. The distribution of the data will be assessed with the Shapiro-Wilk test. Between-group comparisons at baseline will be performed with Student’s t-test, the Mann-Whitney test, or the chi-squared test according to the nature and distribution of the data. Between-group differences in all outcomes according to the trial hypotheses will be tested with Student’s t-test or the Mann-Whitney U test as appropriate. Within-group outcomes will be assessed using the paired-samples t-test or the Wilcoxon signed-rank test. In case of baseline differences between groups, appropriate adjustment measures (e.g., analysis of covariance) will be carried out. An intention-to-treat analysis will be used, with missing values replaced using the multiple imputation procedure (although all efforts will be made to minimize their incidence). A sensitivity analysis (e.g., per protocol) will also be performed to test the stability of the conclusions.

Moreover, Kaplan–Meier analysis and Cox regression will be used to model the incidence rates of long-term clinical outcomes (hospitalization and death). Subgroup analyses will be scheduled to determine the impact of the findings in smaller populations (e.g., to determine gender differences [40], etc.). Kaplan-Meier estimates of the distribution of times from the beginning of rehabilitation to the clinical events and a log-rank test for trend will be used to compare the curves of the patient groups. A p value < 0.05 will be considered significant. Data will be analyzed using SPSS 21.0 (IBM, Armonk, NY, USA) and MedCalc statistical software (version 19.8 and later, MedCalc Software bvba, Ostend, Belgium).

### 2.13. Ethics and Dissemination

The trial has been approved by the Area-Vasta Emilia-Romagna Centro Ethics Committee with approval number 898/20. Written informed consent will be obtained from all participants. Upon conclusion of the trial, the research coordinator will be responsible for the final dataset that will be published in a public repository and made accessible to researchers.

The results of the present study will be published in peer-reviewed journals and presented at national and international congresses. Moreover, the trial reports and publications will be made available to local stakeholders and policymakers and shared within patients’ associations.

### 2.14. Patient and Public Involvement

The study protocol, including intervention and outcome measures, was conceived without patients or public involvement. However, patients will be considered as a route to disseminate the research findings by word of mouth to other patients, especially within patients’ associations. General practitioners will also be involved in the dissemination of the study results, especially among their patients.

## 3. Conclusions

In conclusion, this study may fill in a piece of missing information regarding exercise trials in PAD patients with claudication, addressing some research priorities for exercise interventions recently reported by experts [5]. Leading scientists are indeed advocating studies concerning tailored therapy for different patient abilities; comparing exercise training modalities to maximize adherence and participation; and identifying optimal strategies to transfer patients from supervised, hospital-based settings to community-based settings. Given that a recent trial highlighted the non-superior effect of low-intensity exercise interventions compared to controls in terms of walking ability over 12 months [19], we believe that a different, structured pain-free model of home-based exercise may positively impact the functional capacity and quality of life of claudication patients. In the event of positive results, this program may also be extended to fragile patients with a reduced exercise capacity or with a high degree of PAD severity. The validation of a sustainable and effective patient-centered exercise program compared to usual care may represent a strategic issue for the Health Service to reduce revascularizations, physical decline and hospitalizations in patients with PAD as well as in elderly or frail individuals.

## Figures and Tables

**Table 1:** Weekly training progression for patients randomized into TiTo-SHB group.

Week	Walk:Rest Ratio (min)	Repetitions	Speed Steps/min–km/h
1	1:1	8	60–1.5
2	1:1	8	60–1.5
3	1:1	8	63–1.7
4	1:1	8	63–1.7
5	1:1	8	66–1.9
6	1:1	8	66–1.9
7	1:1	8	69–2.2
8	1:1	8	72–2.4
9	1:1	8	72–2.4
10	1:1	8	76–2.7
11	1:1	8	76–2.7
12	1:1	8	80–3.0
13	1:1	8	76–2.7
14	1:1	8	80–3.0
15	1:1	8	84–3.2
16	1:1	8	84–3.2
17	1:1	8	88–3.5
18	1:1	8	88–3.5
19	1:1	8	92–3.8
20	1:1	8	84–3.2
21	1:1	8	88–3.5
22	1:1	8	92–3.8
23	1:1	8	96–4.0
24	1:1	8	100–4.2

**Table 2:** Outcome measures flow diagram. The line represents the duration of the treatments.

	Study Period			
	Enrollment	Allocation	Post-Allocation	Close-Out
Time point	−15 days (maximum)	0 Baseline	6-month end of training	12-month
Eligibility screening	X			
Informed consent	X			
Allocation		X		
Randomization		X		
Interventions				
Structured Home Based Exercise		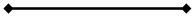		
Control (walking advice)		X	X	
Outcomes				
Pain-free walking distance [primary outcome]		X	X	
6-min walking distance, lower limbs strength, quality of life, foot temperature [secondary]		X	X	
Hospitalizations and mortality			X	X

## Data Availability

Not applicable.
